# ONECUT2 as a key mediator of androgen receptor-independent cell growth and neuroendocrine differentiation in castration-resistant prostate cancer

**DOI:** 10.20517/cdr.2021.108

**Published:** 2022-02-08

**Authors:** WonSeok William Choi, Julia L. Boland, Jianqing Lin

**Affiliations:** Division of Hematology/Oncology and Department of Medicine, George Washington University Hospital, George Washington University School of Medicine and Health Sciences, Washington, DC 20037, USA.

**Keywords:** Prostate cancers, castration resistant, ONECUT2, androgen receptor-independence, neuroendocrine differentiation

## Abstract

Despite androgen dependence in a majority of castration-resistant prostate cancers, some cancer cells are independent of androgen receptor (AR) function, a feature of heterogeneity in prostate cancer. One of the aggressive variants of prostate cancer that are AR independent is neuroendocrine prostate cancer (NEPC). This manuscript will focus on the new finding of human one cut domain family member 2 (ONECUT2) transcription factor and its role in castration resistance, especially in NEPC.

## INTRODUCTION

Prostate cancer is the most common cancer diagnosed in men and the second leading cause of cancer-related deaths in the United States^[1]^. Given its dependence on the AR axis, prostate adenocarcinoma can respond to androgen deprivation therapy (ADT) for a variable duration, but eventually, it progresses to metastatic castration-resistant prostate cancer (mCRPC)^[2]^. Most recently, the newer generation of androgen receptor (AR) signaling inhibitors such as enzalutamide and apalutamide has improved outcomes in patients with mCRPC, but they inevitably develop resistance to these drugs as well. There are several proposed mechanisms of resistance to androgen deprivation or AR inhibitors, many of which are thought to be AR-dependent, including AR-activating mutations and constitutively active AR splice variants^[2]^. However, there are also AR-independent mechanisms, with very low or absent AR expression found in tumor cells that render ADT ineffective and are associated with neuroendocrine (NE) differentiation^[2]^. NEPC is an aggressive variant of prostate cancer that exhibits not only AR independence but also neuroendocrine (NE) differentiation and even distinct histological features such as small cell carcinoma instead of adenocarcinoma^[2,3]^. Though NEPC can rarely arise *de novo*, more commonly, it arises from adenocarcinoma in response to selection pressure for AR-independent cells from treatment with AR signaling inhibitors via lineage plasticity and NE differentiation^[3,4]^. Recent studies spearheaded by Rotinen *et al*.^[5]^ and Guo *et al*.^[6]^ showed that ONECUT2 is an important regulator of AR-mediated growth and a driver of NE differentiation in transition from adenocarcinoma to NEPC, and is being investigated as a new drug target with potential therapeutic implications. This manuscript explains how ONECUT2 leads to AR independence and NE differentiation observed in NEPC.

## ONECUT2 AND PROSTATE CANCER

ONECUT is a family of transcription factors related to hepatic nuclear factor 6, which has been shown to be involved in organogenesis, cell fate, and tumorigenesis^[7]^. In particular, ONECUT1 plays an important role in hepatobiliary tract disease by regulating the development, differentiation, and function of hepatocytes and cholangiocytes^[7]^. Decreased expression of ONECUT1 and its target genes is associated with malformation of the liver, bile duct, gallbladder, and pancreas, as well as diabetes. Furthermore, ONECUT1 may also play a role in the prevention of hepatocellular carcinoma and pancreatic cancer by acting as a tumor suppressor^[7]^. In addition, ONECUT1 also helps regulate the development of retina and motor neuron^[7]^.

ONECUT2 is another transcription factor that regulates cell proliferation, migration, and differentiation, which was first discovered by Jacquemin *et al*.^[8]^ in 1999. ONECUT2 is found in various organs, including the liver, skin, brain, testis, and bladder^[8]^. In contrast to ONECUT1, ONECUT2 expression has been shown to be elevated in multiple different cancers, including prostate cancer^[6]^, ovarian cancer^[9]^, gastric cancer^[10]^, colorectal cancer^[11]^, hepatocellular carcinoma^[12]^, lung adenocarcinoma^[13]^, and neuroendocrine tumors^[6]^. Specifically in prostate cancer, increased ONECUT2 expression has been linked to the aggressiveness of the disease, disease progression, biochemical recurrence, and metastasis^[5,6]^. ONECUT2 mRNA level was found to be elevated in mCRPC prior to any treatment and was higher than in non-metastatic tumors^[5]^. ONECUT2 target genes are involved in the cell cycle, angiogenesis, and hypoxia, which in turn are implicated in tumor growth and metastasis in prostate cancer^[6]^.

Both ONECUT1 and ONECUT2 are transcriptional activators of the ONECUT family with similar but distinct functions in development and pathogenesis in various organs. Similarities and differences between ONECUT1 and ONECUT 2 are outlined in Table 1.

**Table 1:** Characteristics of ONECUT1 and ONECUT2

	**ONECUT1**	**ONECUT2**
Location of gene	15q21.3	18q21.31
Target gene	- Liver genes, including hepatocyte nuclear factor (Hnf) - FOXA1/2 - Transthyretin gene - Glucokinase (Gck), glucose transporter 2 (Glut2) - miR-122	- Liver genes, including hepatocyte nuclear factor (Hnf) - FOXA1/2 - Transthyretin gene - Genes regulated by AR
Functions in development	- Expressed in retinal progenitor cells - Important for the development of liver, bile duct, pancreas - Associated with malformation of the hepatobiliary tract, maturity onset diabetes of the young	- Expressed in retinal progenitor cells - Melanocyte and hepatocyte differentiation
Associated cancers	- Pancreatic cancer - Hepatocellular carcinoma	- Prostate cancer - Ovarian cancer - Gastric cancer - Colorectal cancer - Hepatocellular carcinoma - Lung adenocarcinoma - Neuroendocrine tumors
Ref.	[7]	[7-13]

### ONECUT2 mediates AR independence in prostate cancer

Rotinen *et al*.^[5]^ showed that ONECUT2 expression is negatively correlated to AR activity. AR activity was significantly lower in mCRPC tumors with high ONECUT2 expression, whereas in tumors with high AR activity, the ONECUT2 expression was suppressed^[5]^. ONECUT2 directly suppressed genes regulated by AR, including kallikrein-related peptidase 3 (KLK3)/prostate-specific antigen, kallikrein-related peptidase 2 (KLK2), and ETS homologous factor^[5]^. Altogether, these findings show that ONECUT2 leads to AR independence in prostate cancer. Tumors with high ONECUT2 expression such as NEPC represent a variant group of mCRPC that is independent of AR function.

### ONECUT2 promotes NE differentiation in prostate cancer

**Figure 1:**
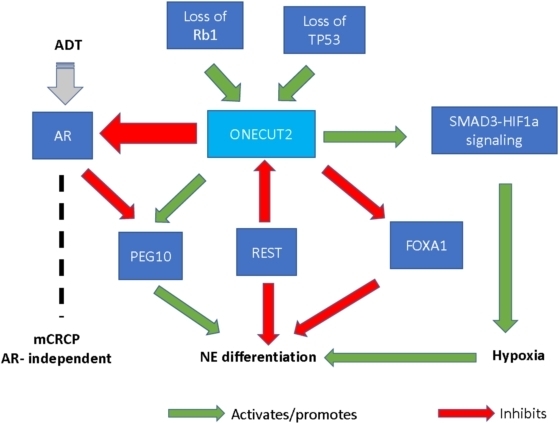
Role of ONECUT2 in AR independence and NE differentiation. ADT: Androgen deprivation therapy; NE: neuroendocrine; mCRPC: metastatic castration-resistant prostate cancer; ONECUT2: one cut domain family member 2; REST: RE1-silencing transcription factor; AR: androgen receptor; PEG10: paternally expressed gene 10; Rb1: retinoblastoma gene 1; FOXA1: forkhead box A1; TP53: p53-encoding gene; SMAD3: SMAD family member 3; HIF1α: hypoxia-inducible factor 1 alpha.

NE differentiation in prostate cancer is associated with a more aggressive phenotype, metastatic disease, and poor response to AR signaling inhibitors^[3]^. ONECUT2 has been shown to play a role in NE differentiation in prostate cancer. A significantly higher level of ONECUT2 expression was found in NEPC compared to adenocarcinoma, and conversely, a reduction in ONECUT2 expression was shown to decrease NE marker gene expression^[5]^. Deletion of TP53 and Rb1, two of the most frequently mutated genes in NEPC, was shown to increase ONECUT2 expression and promote NE plasticity in prostate adenocarcinoma^[6]^. ONECUT2 upregulates genes involved in NE differentiation, such as neuron-specific enolase, synaptophysin, and chromogranin A^[2]^. Furthermore, ONECUT2 has complex interactions with modulators of NE differentiation such as RE1-silencing transcription factor (REST), forkhead box A1 (FOXA1), and paternally expressed gene 10 (PEG10)^[5,14,15]^. REST is an inhibitory regulator of NE differentiation that directly suppresses ONECUT2^[5]^. A decrease in REST expression leads to upregulation of ONECUT2 mRNA and allows for the transition from adenocarcinoma to NEPC^[5]^. FOXA1, another modulator that normally inhibits NE differentiation, is suppressed by ONECUT2 during transdifferentiation of adenocarcinoma into NEPC^[5]^. PEG10 is different from REST and FOXA1 in that it promotes NE differentiation and is directly suppressed by AR^[5,15]^. An increase in ONECUT2 expression corresponds to upregulation of PEG10 and transition from adenocarcinoma to NEPC^[5]^. Figure 1 demonstrates the role of ONECUT2 in AR independence and NE differentiation.

## ONECUT2 REGULATES HYPOXIA SIGNALING, WHICH PROMOTES NE DIFFERENTIATION IN PROSTATE CANCER

Guo *et al*.^[6]^ demonstrated that hypoxia can induce NE differentiation and disease progression in prostate cancer, evidenced by an increase in NE marker gene expression in hypoxic conditions compared to normoxic ones. Knockdown of hypoxia-inducible factor 1α (HIF1α), a transcription factor that regulates the hypoxia signaling pathway, led to a reduction in NE marker gene expression^[6]^. Guo *et al*.^[6]^ also showed that ONECUT2 is involved in regulating the hypoxia pathway, with ONECUT2 activity correlating with tumor hypoxia in the progression from adenocarcinoma to NEPC. ONECUT2 upregulates hypoxia-associated genes such as adrenomedullin and angiopoietin-like 4 by activating SMAD family member 3, which interacts with HIF1α in regulating hypoxia signaling^[6]^ (see Figure 1). Altogether, these findings suggest that regulating the hypoxia pathway is yet another way how ONECUT2 contributes to NE differentiation.

## ONECUT2 AS A DRUG TARGET IN PROSTATE CANCER

Following the discovery of ONECUT2 and its role in the transition from prostate adenocarcinoma to NEPC, new drugs targeting this pathway are being developed and studied in clinical trials. CSRM617, a small molecule inhibitor of ONECUT2 developed by Rotinen *et al*.^[5]^, led to cell death in prostate cancer cell lines with high ONECUT2 expression. The level of ONECUT2 expression was positively correlated with treatment response to CSRM617^[5]^. *In vivo*, CSRM617 led to significant reduction in tumor size, PEG10 protein level (a marker of NE differentiation), and metastatic growth^[5]^.

TH-302 (evofosfamide) is a hypoxia-activated prodrug with an alkylating agent moiety that is released only in hypoxic environments, such as those found in hypoxic tumors like NEPC^[16]^. TH-302 was shown to significantly inhibit tumor growth in NEPC, but less so in adenocarcinoma^[6]^. There was a positive correlation between the level of ONECUT2 expression and the treatment response to TH-302^[6]^. These findings show that NEPC and a higher level of ONECUT2 expression are both associated with a greater degree of hypoxia, which in turn lead to an enhanced response to TH-302. TH-302 is being studied in clinical trials, including a phase I/II clinical trial (NCT00743379) that focuses on the efficacy of TH-302 in combination with chemotherapy such as docetaxel in multiple solid tumors, including mCRPC that were not previously treated with chemotherapy. Another phase I study (NCT03098160) examines the safety and toxicity of TH-302 in combination with ipilimumab in advanced solid malignancies, including mCRPC.

## FUTURE DIRECTIONS

Given the rising use of newer generation AR signaling inhibitors, mCRPC exhibiting AR independence will likely grow in prevalence^[2]^. Treatment of prostate cancer with AR inhibition may lead to selection for cancer cells with elevated ONECUT2 expression, thereby contributing to resistance to AR-targeted therapy and development of NE features that lead to more aggressive phenotypes with worse clinical outcomes^[2,3,5]^. It is, therefore, crucial to continue further research on the understanding of the master regulators of AR independence such as ONECUT2 and develop safe and efficacious drugs against such targets. We believe biomarker-driven correlative studies are key for the success of future drug development targeting ONECUT2.

## DECLARATIONS

### Copyright

© The Author(s) 2022.

## References

[1] Siegel RL, Miller KD, Fuchs HE, Jemal A (2021). Cancer statistics, 2021. CA Cancer J Clin.

[2] Watson PA, Arora VK, Sawyers CL (2015). Emerging mechanisms of resistance to androgen receptor inhibitors in prostate cancer. Nat Rev Cancer.

[3] Aggarwal R, Zhang T, Small EJ, Armstrong AJ (2014). Neuroendocrine prostate cancer: subtypes, biology, and clinical outcomes. J Natl Compr Canc Netw.

[4] Rickman DS, Beltran H, Demichelis F, Rubin MA (2017). Biology and evolution of poorly differentiated neuroendocrine tumors. Nat Med.

[5] Rotinen M, You S, Yang J (2018). ONECUT2 is a targetable master regulator of lethal prostate cancer that suppresses the androgen axis. Nat Med.

[6] Guo H, Ci X, Ahmed M (2019). ONECUT2 is a driver of neuroendocrine prostate cancer. Nat Commun.

[7] Kropp PA, Gannon M (2016). Onecut transcription factors in development and disease. Trends Dev Biol.

[8] Jacquemin P, Lannoy VJ, Rousseau GG, Lemaigre FP (1999). OC-2, a novel mammalian member of the ONECUT class of homeodomain transcription factors whose function in liver partially overlaps with that of hepatocyte nuclear factor-6. J Biol Chem.

[9] Lu T, Wu B, Yu Y (2018). Blockade of ONECUT2 expression in ovarian cancer inhibited tumor cell proliferation, migration, invasion and angiogenesis. Cancer Sci.

[10] Chen J, Chen J, Sun B, Wu J, Du C (2020). ONECUT2 accelerates tumor proliferation through activating ROCK1 expression in gastric cancer. Cancer Manag Res.

[11] Sun Y, Shen S, Liu X (2021). Correction to: miR-429 inhibits cells growth and invasion and regulates EMT-related marker genes by targeting Onecut2 in colorectal carcinoma. Mol Cell Biochem.

[12] Zhang J, Cheng J, Zeng Z (2015). Comprehensive profiling of novel microRNA-9 targets and a tumor suppressor role of microRNA-9 via targeting IGF2BP1 in hepatocellular carcinoma. Oncotarget.

[13] Ma Q, Wu K, Li H (2019). ONECUT2 overexpression promotes RAS-driven lung adenocarcinoma progression. Sci Rep.

[14] Lapuk AV, Wu C, Wyatt AW (2012). From sequence to molecular pathology, and a mechanism driving the neuroendocrine phenotype in prostate cancer. J Pathol.

[15] Akamatsu S, Wyatt AW, Lin D (2015). The placental gene PEG10 promotes progression of neuroendocrine prostate cancer. Cell Rep.

[16] Meng F, Evans JW, Bhupathi D (2012). Molecular and cellular pharmacology of the hypoxia-activated prodrug TH-302. Mol Cancer Ther.

